# Solitary Fibrous Tumor Arising in the Buccal Space

**DOI:** 10.1155/2019/9459837

**Published:** 2019-10-09

**Authors:** Tatsuo Okui, Soichiro Ibaragi, Hotaka Kawai, Akira Sasaki

**Affiliations:** ^1^Department of Oral and Maxillofacial Surgery, Okayama University Graduate School of Medicine, Dentistry and Pharmaceutical Sciences, Okayama, Japan; ^2^Department of Oral Pathology and Medicine, Okayama University Graduate School of Medicine, Dentistry and Pharmaceutical Sciences, Okayama, Japan

## Abstract

A 39-year-old Japanese woman presented to the Department of Oral and Maxillofacial Surgery, Okayama University Hospital, with the complaint of a slowly growing buccal mass. The mass was well defined, had rounded margins, and was free from skin and muscles. A color Doppler echographic examination indicated high flow velocity of the blood surrounding the mass. Contrast-enhanced images on CT and contrast-enhanced T1-weighted images on MRI displayed a homogeneous enhanced mass with a well-defined margin. A fine-needle aspiration biopsy and histological examination were performed. On immunohistochemistry, spindle cells were strongly positive for CD34, STAT6, and vimentin and negative for EMA, S100, and *α*-SMA. The tumor was removed with extracapsular dissection. The tumor was composed of bland spindle cells proliferating in a patternless arrangement with a collagenous background. Most of the tumor mass consisted of hypocellular areas including ectatic blood vessels. A prominent branching vascular pattern was observed. Immunohistochemistry demonstrated that the tumor cells were positive for CD34, STAT6, vimentin, and Bcl-2, and negative for *α*-SMA, S100, and EMA. Three mitotic cells were observed per 10 high-power fields, and the Ki-67 index was 5.7%. The morphological and immunohistochemical features were consistent with a diagnosis of solitary fibrous tumor.

## 1. Case Report

**Figure 1:**
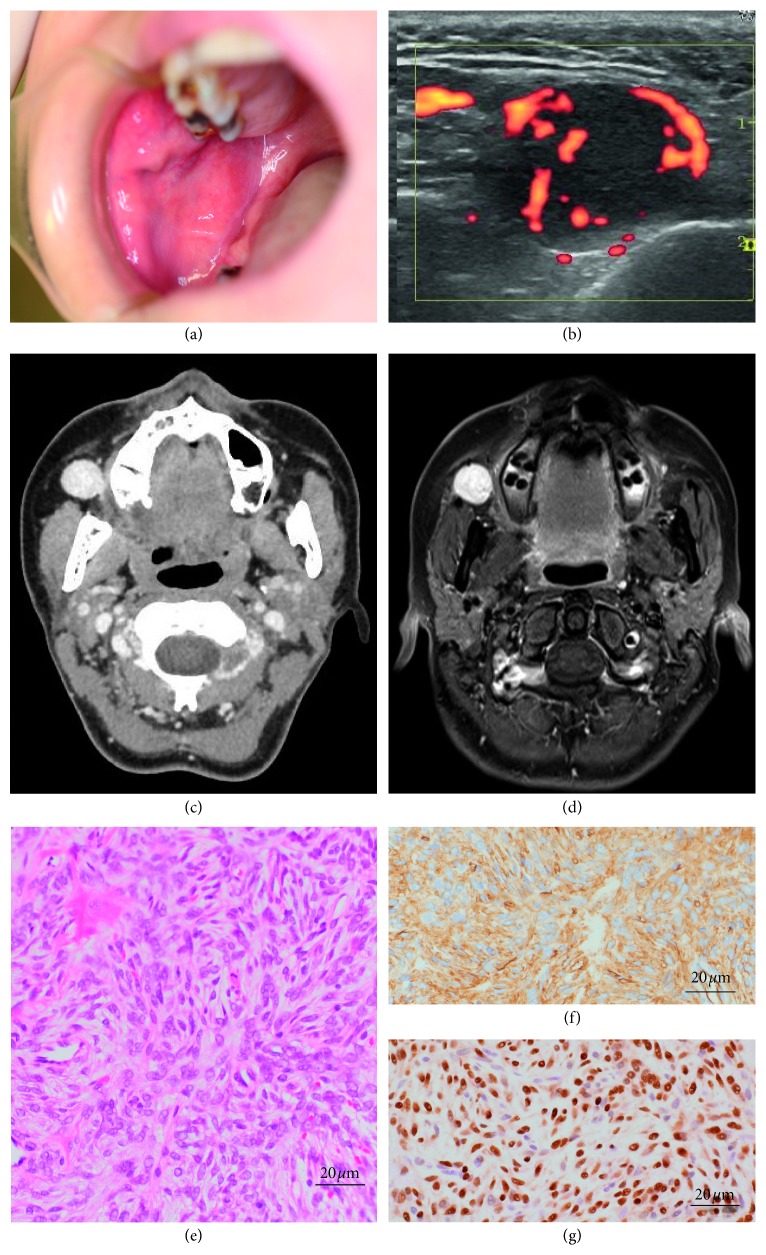
(a) Intraoral photograph: a painless submucosal mass in the right buccal region. (b) Doppler ultrasonography of the buccal mass. High blood flow was observed around the nodular mass with low echogenicity. (c) Horizontal CT images. (d) MR images. T1-weighted imaging. (e) Tumor cells were arranged in a patternless manner. Variation in the size and shape of the cells or their nuclei was not conspicuous (FNAB hematoxylin/eosin staining). (f) Tumor cells showed diffuse positivity for CD34 (FNAB IHC) and (g) nuclear positivity for STAT6 (FNAB IHC).

A 39-year-old Japanese woman presented to the Department of Oral and Maxillofacial Surgery at Okayama University Hospital with the complaint of a buccal mass that had been growing slowly for 3 years. There were no medical records regarding the mass. The clinical examination revealed a 1.5 × 1.5 cm round mass at the buccal space. The mass was well defined with rounded margins and free from skin and muscles (Figure 1(a)). There were no palpable lymph nodes in the neck. The mass did not elicit pain.

A color Doppler echographic examination indicated high flow velocity of the blood surrounding the mass (Figure 1(b)). A contrast-enhanced image on computed tomography (Figure 1(c)) and contrast-enhanced T1-weighted image on magnetic resonance imaging (Figure 1(d)) displayed 1.5 × 1.5 cm homogeneous enhanced mass in the front of the masseter muscle with a well-defined margin. We performed a fine-needle aspiration biopsy (FNAB). The major part of the corrected cell block specimen obtained by the FNAB was spindle cells. These spindle cells were arranged and lined in a patternless manner, and variation in the size and shape of the cells or their nuclei was not conspicuous (Figure 1(e)). On immunohistochemistry, the spindle cells were strongly positive for CD34 (Figure 1(f)), STAT6 (Figure 1(g)), and vimentin and negative for EMA, S100, and *α*-SMA.

Surgery was conducted with the patient under general anesthesia. A 3.0 cm incision at the right buccal mucosa was made parallel to the anterior border of the mandible ramus. The identified anatomic layers included the mucosa and the buccinator muscle. The tumor was found adjacent to the front part of the buccinator muscle. The tumor was encapsulated with connective tissue. It was easily separated from the layer structure. The tumor was ablated with extracapsular dissection. The parotid gland duct was excised, and the duct orifice was expanded to the buccal mucosa. The patient was discharged 4 days after the surgery. There have been no signs of facial nerve injury or recurrence at 12 months postoperatively.

**Figure 2:**
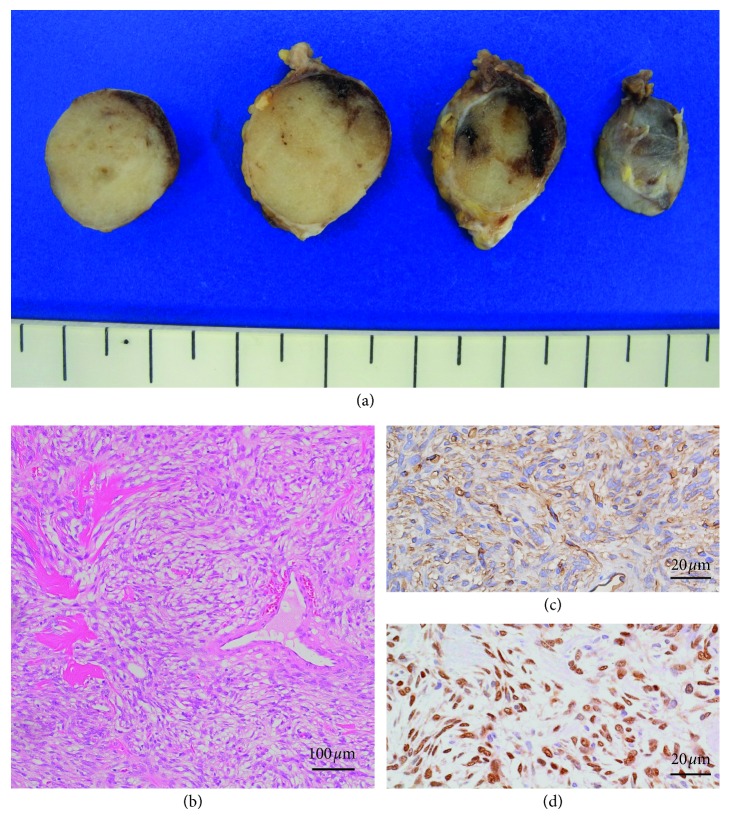
(a) Gross appearance of the resected specimen. A well-circumscribed nodular mass measuring 15 × 15 × 15 mm with a pale or uniformly white cut surface was observed. (b) The tumor comprised spindle cells with an irregular disposition associated with collagen bands and vascular structures branched with an evident lumen. (c) Tumor cells showed strong positivity for CD34 (IHC). (d) Nuclear positive reaction for STAT6 in spindle tumor cells (IHC).

Macroscopically, the cut section of the resected specimen showed a circumscribed pale or uniformly white mass measuring 15 × 15 × 15 mm surrounded by a fibrous capsule (Figure 2(a)). Microscopy revealed that the tumor was composed of bland spindle cells proliferating in a patternless arrangement with a collagenous background. Most of the tumor mass consisted of hypocellular areas including ectatic blood vessels (Figure 2(b)).

A prominent branching vascular pattern was observed. Immunohistochemistry (IHC) demonstrated that the tumor cells were positive for CD34 (Figure 2(c)), STAT6 (Figure 2(d)), vimentin, and Bcl-2 and negative for *α*-SMA, S100, and EMA. Three mitotic cells were observed per 10 high-power fields (HPFs), and the Ki-67 index was 5.0%. The morphological and immunohistochemical features were consistent with the diagnosis of solitary fibrous tumor.

## 2. Discussion

Solitary fibrous tumor (SFT) is a rare, mesenchymal tumor that usually originates from the pleura and peritoneum [1]. SFT as an oral or maxillofacial lesion is extremely rare, and the behavior of SFTs at this location is not clearly understood [2]. SFT is categorized as an intermediate fibroblastic tumor in the World Health Organization (WHO) classification [3]. SFTs do not show characteristic images on CT or MRI [4]. The diagnosis of SFT thus depends on the findings obtained in a histological examination.

SFTs show distinctive histologic features including fibroblast-like cells with a patternless arrangement, allowing for a definitive diagnosis without the need for special stains at a particular site [5]. However, for SFTs at less common sites, the diagnosis is often challenging, particularly when only a small biopsy specimen is available or there is a less characteristic histologic pattern [6]. In the present case, a cell block could be collected with an 18-gauge fine needle, and the block was evaluated immunohistochemically with antibodies of CD34, Ki-67, vimentin, S100, and STAT6. The Ki-67 expression was <6%, and there were no necrotic areas. These histopathological features indicated aspects of a benign tumor.

We found only two cases of SFT at the buccal space that underwent a preoperative FNAB [7, 8]. In both cases, the FNAB results were nonspecific granulation tissue. Fortunately, the large cell block that was collected by the FNAB in the present patient's case led to the correct diagnosis.

A useful discovery from a whole-exome sequencing study revealed that NAB2-STAT6 fusion is characteristic of SFTs [9]. In our patient's case, strong STAT6 expression was observed and indicated an SFT. However, STAT6 expression is not a specific marker because STAT6 is also expressed in a small percentage of desmoid tumors and unclassified sarcomas that could be confused with SFT [10]. The 2013 WHO classification of soft tissue tumors defines malignant forms as hypercellular, mitotically active (>4 mitoses/10 HPF), with cytological atypia, tumor necrosis, and infiltrative margins [11]. In the present case, the immunohistological features demonstrated by the FNAB indicated no malignant forms of SFT. Based on the preoperative diagnosis, we performed a capsulized tumor resection. FNAB is useful for the diagnosis of SFTs in the oral and maxillofacial region.

The recurrence rate of SFT occurring in the pleura is reported to be approx. 30% [12]. In contrast, recurrence of an SFT in an oral lesion is rare [13]. Expanding the scope to include head and neck lesions increases the recurrence rate to 40% [14]. Some reports indicated that late relapses (>10 years from the first diagnosis) can occur in head and neck SFTs [14]. More importantly, clinicians should keep in mind that a patient with a past history of SFT can show a malignant relapse even when the pathological features indicated a benign SFT in the first diagnosis. Thus, a continuous long-term follow-up is needed for SFT patients.

## References

[1] Hanau C., Miettinen M. (1995). Solitary fibrous tumor: histological and immunohistochemical spectrum of benign and malignant variants presenting at different sites. *Human Pathology*.

[2] Raghani M., Raghani N., Rao S., Rao S. (2018). Hemangiopericytoma/solitary fibrous tumor of the buccal mucosa. *Annals of Maxillofacial Surgery*.

[3] Fletcher C. D. M. (2006). The evolving classification of soft tissue tumours: an update based on the new WHO classification. *Histopathology*.

[4] Liu Y., Tao X., Shi H., Li K. (2014). MRI findings of solitary fibrous tumours in the head and neck region. *Dentomaxillofacial Radiology*.

[5] You Y.-H., Liu R.-T., Zhang Y. (2018). A large solitary fibrous tumour of the pleura: a case report and review of the literature. *Journal of International Medical Research*.

[6] Gupta N., Barwad A., Katamuthu K. (2012). Solitary fibrous tumour: a diagnostic challenge for the cytopathologist. *Cytopathology*.

[7] Dunfee B. L., Sakai O., Spiegel J. H., Pistey R. (2005). Solitary fibrous tumor of the buccal space. *American Journal of Neuroradiology*.

[8] Künzel J., Hainz M., Ziebart T. (2016). Head and neck solitary fibrous tumors: a rare and challenging entity. *European Archives of Oto-Rhino-Laryngology*.

[9] Robinson D. R., Wu Y.-M., Kalyana-Sundaram S. (2013). Identification of recurrent NAB2-STAT6 gene fusions in solitary fibrous tumor by integrative sequencing. *Nature Genetics*.

[10] Tan S. Y., Szymanski L. J., Galliani C., Parham D., Zambrano E. (2018). Solitary fibrous tumors in pediatric patients: a rare and potentially overdiagnosed neoplasm, confirmed by STAT6 immunohistochemistry. *Pediatric and Developmental Pathology*.

[11] Fletcher C. D. M., Lee J. C. (2013). Extrapleural solitary fibrous tumor. *World Health Organization Classification of Tumours of Soft Tissue and Bone*.

[12] Lococo F., Cesario A., Cardillo G. (2012). Malignant solitary fibrous tumors of the pleura: retrospective review of a multicenter series. *Journal of Thoracic Oncology*.

[13] Alawi F., Stratton D., Freedman P. D. (2001). Solitary fibrous tumor of the oral soft tissues: a clinicopathologic and immunohistochemical study of 16 cases. *The American Journal of Surgical Pathology*.

[14] Smith S. C., Gooding W. E., Elkins M. (2017). Solitary fibrous tumors of the head and neck: a multi-institutional clinicopathologic study. *The American Journal of Surgical Pathology*.

